# Correlates of Full, Partial, and Non‑Immunisation among Children Aged 12–23 Months in Gokwe South District, Rural Zimbabwe

**DOI:** 10.5334/aogh.5209

**Published:** 2026-06-24

**Authors:** Laston Gonah, Norest Hama, Olanrewaju Oladimeji

**Affiliations:** 1 School of Public Health, Faculty of Medicine and Health Sciences, iYunivesithi Walter Sisulu, Mthatha, 5100, South Africa; 2 Department of Applied Mathematics and Statistics, Faculty of Science and Technology, Midlands State University, Gweru, Zimbabwe; 3 Faculty of Life Sciences and Education, University of South Wales, Cardiff, CF24 2FN, United Kingdom; 4 Department of Public Health, Sefako Makgatho Health Sciences University, Pretoria, South Africa; 5 SAMRC/SMU Public Health Interventions, Innovations and Implementation Research Unit, Sefako Makgatho Health Sciences University, Pretoria, South Africa

**Keywords:** childhood immunisation, vaccine uptake, Zimbabwe, logistic regression, maternal factors, rural health, Gokwe District

## Abstract

Childhood immunisation coverage is often lower in rural and hard-to-reach areas of Zimbabwe than national targets, potentially leaving children vulnerable to vaccine-preventable diseases. We examined factors associated with immunisation status among 12–23-month-old children in rural Zimbabwe, using a cross-sectional study among caregivers of 12–23-month-old children. These were selected using a multistage sampling strategy, with households randomly selected within health facility catchment areas. Logistic regression models identified correlates of incomplete immunisation. Among 573 caregiver–child pairs (54.8% male), 62.1% of children were fully immunised, 24.6% partially immunised, and 13.3% not immunised; coverage did not differ by sex (*p* = 0.12). Bivariate analyses showed significant associations with possession of a child health card, maternal education, place of birth, religious affiliation, knowledge of immunisation schedules, and caregiver satisfaction with healthcare (*p* < 0.05). In the adjusted binary logistic regression model, caregiver dissatisfaction (AOR = 2.6; 95% CI: 1.7–4.0), absence of a child health card (AOR = 3.5; 95% CI: 2.2–5.5), limited knowledge of new vaccines (AOR = 1.9; 95% CI: 1.2–3.1), and apostolic affiliation (AOR = 0.44; 95% CI: 0.26–0.73) independently predicted non-immunisation. Multinomial regression showed that home birth, low maternal knowledge, and lack of health cards differentiated partial from complete non-immunisation. Improving caregiver health literacy, strengthening health-system responsiveness, ensuring continuity of child health records, and engaging apostolic religious communities may substantially improve childhood immunisation coverage in rural Zimbabwe.

## Introduction

Vaccination has substantially reduced childhood morbidity and mortality worldwide and is considered one of the most cost‑effective public health interventions. Despite global progress, regions within low‑ and middle‑income countries (LMICs) continue to experience low immunisation coverage, leaving children vulnerable to vaccine‑preventable diseases (VPDs) [1]. Sub‑Saharan Africa remains disproportionately affected, with persistent inequities between urban and rural populations [2–4].

In Zimbabwe, routine childhood vaccines are provided free of charge through public health facilities under the Expanded Programme on Immunisation (EPI) [5, 6]. The country has historically achieved high levels of routine immunisation coverage; however, pockets of suboptimal uptake persist in rural districts. Gokwe District, which is predominantly rural, geographically expansive, and characterised by high poverty levels, is among the areas where challenges in vaccination delivery persist [5]. Understanding the correlates of full, partial, and non‑immunisation is essential for achieving national and global targets, including the Immunization Agenda 2030 and Sustainable Development Goal 3 [4, 7, 8].

Previous studies in Zimbabwe and comparable settings have emphasised the roles of maternal knowledge, distance to health facilities, socio‑economic status, place of delivery, health system bottlenecks, and socio‑cultural beliefs in shaping immunisation behaviours [5, 9–12]. However, limited work has simultaneously modelled complete, partial, and non‑immunisation outcomes within a single analytic framework.

This study therefore employs binary and multinomial logistic regression models to identify socio‑demographic, maternal, health system, and knowledge‑based correlates of low childhood immunisation uptake in Gokwe District. The analysis offers evidence to inform targeted interventions for improving routine immunisation performance in rural Zimbabwe.

## Methods

### Study design and setting

This was a community‑based cross‑sectional study conducted in Gokwe District, a predominantly rural district in the Midlands Province of Zimbabwe [13, 14]. The district is characterised by dispersed settlements, limited transportation networks, high poverty, and reliance on subsistence farming [13]. Health services are delivered through clinics, rural hospitals, and outreach points supported by village health workers.

### Study population

The study population comprised caregivers of children aged 12–23 months, residing in Gokwe South District for at least 6 months prior to the survey. Children younger than 12 months were excluded to avoid misclassification of incomplete schedules.

### Sampling procedure

A multistage sampling approach was employed. In the first stage, health facilities providing routine childhood immunisation services in the study area were selected purposively to ensure representation of the major immunisation service delivery points across the district. Within these catchment areas, wards and villages were randomly selected, and households were allocated proportionally according to population size. In the final stage, households with eligible children were selected using systematic sampling. Where more than one eligible child was present in a household, one child was selected using simple random sampling.

The required sample size was estimated using Cochran’s formula [15] for estimating a population proportion for an infinite population, *n* = (*Z*² × *P* (1 − *P*)) / *e*², assuming an immunisation coverage proportion (*P* = 0.5), a 95% confidence level (*Z* = 1.96), and a margin of error of 5%. To account for the multistage sampling design and potential non‑response, the sample size was adjusted using a design effect of 1.2. Ultimately, 573 caregiver–child pairs with complete data consented to participate and were included in the final analysis.

### Data collection

Data were collected using structured questionnaires administered to caregivers. Information obtained included socio‑demographic characteristics, maternal factors, knowledge of immunisation, health service accessibility, and child vaccination status as recorded in the child health card.

Immunisation status was defined according to the Zimbabwe Expanded Programme on Immunisation (ZEPI) [5] schedule in effect during the study period. Children were receiving routine vaccinations from birth through the first year of life as follows: at birth, Bacille Calmette‑Guérin (BCG), oral polio vaccine zero dose (OPV0), and the Hepatitis B birth dose; at 6 weeks of age, the first doses of pentavalent vaccine (DTP‑HepB‑Hib1), oral polio vaccine (OPV1), pneumococcal conjugate vaccine (PCV1), and rotavirus vaccine (Rotavirus1); at 10 weeks, the second doses of pentavalent vaccine (DTP‑HepB‑Hib2), oral polio vaccine (OPV2), pneumococcal conjugate vaccine (PCV2), and rotavirus vaccine (Rotavirus2); at 14 weeks, the third doses of pentavalent vaccine (DTP‑HepB‑Hib3), oral polio vaccine (OPV3), and pneumococcal conjugate vaccine (PCV3); and at 9 months, the measles‑rubella (MR) vaccine [5, 6].

Children were classified as fully immunised if they had received all age‑appropriate vaccine doses recommended under the national schedule by 12 months of age, as verified from the child health card. Children were classified as partially immunised if they had received at least one vaccine dose but had missed one or more scheduled doses required to complete the national immunisation schedule by 12 months of age. Children who had not received any routine childhood vaccine were classified as not immunised.

The dependent variable was immunisation status, categorised as fully immunised, partially immunised, or not immunised; for binary logistic regression, children were classified as fully immunised (1) and incompletely immunised (partial or non‑immunised) (0). Independent variables comprised child factors (age, sex, and birth order); maternal factors (such as age, education, parity, and marital status); household characteristics (such as income, employment status, and family size); and health system‑related factors (including distance to the health facility, vaccine stockouts, waiting time, and availability of outreach services). Knowledge‑related variables were also assessed, focusing on caregivers’ awareness of immunisation schedules, VPDs, and the recommended timing of childhood vaccinations.

### Data analysis

Data were entered into Microsoft Excel 2019 and analysed using Stata version 16 or R version 4.0. Descriptive statistics summarised participant characteristics and immunisation status. Associations between socio‑demographic, household, and caregiver factors and immunisation status were assessed using chi‑square tests and crude odds ratios (ORs) with 95% confidence intervals (CI). Statistical significance was set at *p* < 0.05. Variables significant at *p* < 0.2 in bivariate analyses were included in multivariate binary and multinomial logistic regression models. Model building followed a backward elimination approach, and model fit was evaluated using Hosmer–Lemeshow tests (binary logistic regression) and likelihood ratio tests (multinomial logistic regression). Multicollinearity was assessed using variance inflation factors (VIF < 5 considered acceptable) [16, 17].

### Ethical considerations

Ethical approval for the study was sought from the Midlands State University Institutional Review Board, the Medical Research Council of Zimbabwe (MRCZ), and the Midlands Provincial and Gokwe South District Health Executive authorities. Written informed consent was obtained from all participating caregivers prior to data collection. Participant anonymity and confidentiality were maintained throughout the study. All study procedures were conducted in accordance with the ethical principles outlined in the Declaration of Helsinki [18].

## Results

### Socio‑demographic characteristics

A total of 573 caregiver–child pairs were included in the analysis. Slightly more than half of the children were male (54.8%), while females accounted for 45.2%. Most children were born in health facilities (67.0%), while 33.0% were home births. Maternal education was generally low, with 35% of caregivers having no formal education, 40% primary education, and 25% secondary or higher education. The religious profile of caregivers reflected the district’s predominantly apostolic landscape: 11% belonged to the Marange apostolic sect, 44% to other apostolic groups, and the remaining 45% were affiliated with Pentecostal, Protestant, or Catholic churches. Additional demographic and household characteristics of the study sample are presented in Table 1.

**Table 1:** Socio‑demographic characteristics of caregivers and children (*n* = 573).

VARIABLE	CATEGORY	FREQUENCY (*N*)	PERCENTAGE (%)
Child sex	Male	314	54.8
	Female	259	45.2
Place of birth	Health facility	384	67.0
	Home	189	33.0
Birth order	1	150	26.2
	2–3	220	38.4
	≥4	203	35.4
Maternal education	None	200	35.0
	Primary	229	40.0
	Secondary or higher	144	25.0
Religious affiliation	Marange Apostolic	63	11.0
	Other Apostolic	252	44.0
	Pentecostal/Protestant/Catholic	258	45.0

### Immunisation coverage

Overall, 356 children (62.1%) were fully immunised, 141 (24.6%) partially immunised, and 76 (13.3%) not immunised. Full immunisation was higher among males (57%) than females (43%), but the difference was not statistically significant (*p* = 0.12). Full, partial, and non‑immunisation by child sex and other characteristics are illustrated in Table 2.

**Table 2:** Immunisation status by child and caregiver characteristics (*n* = 573).

CHARACTERISTIC	FULLY IMMUNISED *N* (%)	PARTIALLY IMMUNISED *N* (%)	NOT IMMUNISED *N* (%)	*P*‑VALUE (*X*^2^ TEST)
Child sex				
Male Female	182 (57.9) 174 (67.2)	83 (26.5) 58 (22.4)	49 (15.6) 27 (10.4)	0.12
Place of birth				
Health facility Home	250 (65.1) 106 (56.1)	90 (23.4) 51 (27.0)	44 (11.5) 32 (16.9)	0.01
Child health card				
Yes No	320 (72.3) 36 (19.8)	90 (20.3) 51 (28.1)	30 (7.4) 46 (52.1)	<0.001
Religious affiliation				
Marange Apostolic Other Apostolic Pentecostal/ Protestant/ Catholic	20 (31.7) 110 (43.7) 226 (87.6)	23 (36.5) 88 (35.0) 30 (11.6)	20 (31.7) 54 (21.4) 2 (0.8)	<0.001

### Bivariate analysis of factors associated with immunisation status

Possession of a child health card strongly predicted full immunisation (OR = 3.2; 95% CI: 2.1–4.8; *p* < 0.001). Children of mothers with secondary or higher education were 2.8 times more likely to be fully immunised than children of mothers with no formal education (95% CI: 1.9–4.0; *p* < 0.001). Children from Marange apostolic households had lower uptake (OR = 0.42; 95% CI: 0.26–0.68; *p* < 0.001). Place of birth, birth order, knowledge of the immunisation schedule, exposure to immunisation education, knowledge of new vaccines, knowledge of VPDs, caregiver satisfaction with healthcare, negative perceptions, and worry about side effects were all significant predictors (all *p* < 0.05). Crude ORs are presented in Table 3.

**Table 3:** Crude odds ratios (ORs) for predictors of immunisation status.

PREDICTOR	CATEGORY	OR	95% CI	*P*‑VALUE
Child health card	Yes vs no	3.2	2.1–4.8	<0.001
Maternal education	Secondary+ vs none	2.8	1.9–4.0	<0.001
Religious affiliation	Marange vs others	0.42	0.26–0.68	<0.001
Place of birth	Health facility vs home	1.9	1.3–2.9	0.001
Birth order	≥4 vs 1	0.7	0.4–1.2	0.18
Knowledge of immunisation schedule	Adequate vs poor	2.1	1.5–3.0	<0.001
Exposure to immunisation education	Yes vs no	1.8	1.3–2.6	0.001
Knowledge of new vaccines	Yes vs no	1.9	1.3–2.8	0.001
Knowledge of VPDs	Adequate vs poor	1.6	1.1–2.4	0.02
Satisfaction with healthcare	Satisfied vs unsatisfied	2.5	1.7–3.8	<0.001
Negative perceptions	Yes vs no	0.6	0.4–0.9	0.01
Worry about side effects	Yes vs no	0.7	0.5–1.0	0.04

### Binary logistic regression

After adjusting for confounders, four variables independently predicted non‑immunisation. Children whose caregivers reported lower satisfaction with healthcare had 2.6 times higher odds of being non‑immunised (AOR = 2.6; 95% CI: 1.7–4.0; *p* < 0.001). Possession of a child health card remained protective (AOR = 3.5; 95% CI: 2.2–5.5; *p* < 0.001). Caregiver knowledge of new vaccines predicted full immunisation (AOR = 1.9; 95% CI: 1.2–3.1; *p* = 0.006). Marange apostolic affiliation was associated with lower immunisation odds (AOR = 0.44; 95% CI: 0.26–0.73; *p* = 0.002). Full regression results are shown in Table 4.

**Table 4:** Adjusted binary logistic regression model for non‑immunisation.

PREDICTOR	CATEGORY	ADJUSTED OR (AOR)	95% CI	*P*‑VALUE
Child health card	Yes vs no	3.5	2.2–5.5	<0.001
Knowledge of new vaccines	Yes vs no	1.9	1.2–3.1	0.006
Satisfaction with healthcare	Satisfied vs unsatisfied	2.6	1.7–4.0	<0.001
Religious affiliation	Marange vs others	0.44	0.26–0.73	0.002

### Multinomial logistic regression

Predictors of non‑immunisation (versus fully immunised) included caregiver dissatisfaction with healthcare (RRR = 2.8; 95% CI: 1.8–4.4; *p* < 0.001), home birth (RRR = 2.2; 95% CI: 1.4–3.5; *p* = 0.001), lack of a child health card (RRR = 4.1; 95% CI: 2.5–6.6; *p* < 0.001), and Marange apostolic affiliation (RRR = 0.39; 95% CI: 0.23–0.65; *p* < 0.001). Partial immunisation (versus fully immunised) was predicted by caregiver dissatisfaction with healthcare (RRR = 1.9; 95% CI: 1.2–3.0; *p* = 0.005), absence of a child health card (RRR = 2.8; 95% CI: 1.7–4.7; *p* < 0.001), and limited knowledge of newly introduced vaccines (RRR = 1.7; 95% CI: 1.1–2.6; *p* = 0.02). Notably, place of birth and religious affiliation were not significant for partial immunisation once other factors were adjusted for, suggesting different barriers influence partial versus complete non‑uptake. Taken together, the multinomial model demonstrates that predictors of partial and complete non‑immunisation are not identical, indicating distinct pathways leading to incomplete immunisation. These findings are summarised in Table 5.

**Table 5:** Multinomial logistic regression results for immunisation status.

PREDICTOR	CATEGORY	PARTIAL IMMUNISATION vs FULLY IMMUNISED RRR(95% CI)	*P*‑VALUE	NON‑IMMUNISED vs FULLY IMMUNISED RRR (95% CI)	*P*‑VALUE
Child health card	Yes vs no	2.8 (1.7–4.7)	<0.001	4.1 (2.5–6.6)	<0.001
Knowledge of new vaccines	Yes vs no	1.7 (1.1–2.6)	0.02	1.5 (0.9–2.5)	0.11
Satisfaction with healthcare	Satisfied vs unsatisfied	1.9 (1.2–3.0)	0.005	2.8 (1.8–4.4)	<0.001
Place of birth	Health facility vs home	1.2 (0.7–2.0)	0.48	2.2 (1.4–3.5)	0.001
Religious affiliation	Marange vs others	0.8 (0.4–1.4)	0.46	0.39 (0.23–0.65)	<0.001

## Discussion

This study investigated correlates of full, partial, and non‑immunisation among children aged 12–23 months using binary and multinomial logistic regression models and found that coverage did not differ significantly by sex, but incomplete immunisation was strongly associated with caregiver dissatisfaction with health services, absence of a child health card, low maternal vaccine knowledge, home births, and Marange apostolic church affiliation. Importantly, predictors of partial versus complete non‑immunisation differed, suggesting distinct pathways to incomplete vaccination.

The strong association between possession of a child health card and full immunisation underscores the importance of record‑keeping and continuous follow‑up in ensuring adherence to vaccination schedules. Caregiver knowledge of newly introduced vaccines and immunisation schedules also independently predicted uptake, highlighting maternal health literacy as a critical driver of immunisation behaviour [11, 12, 19]. These findings are consistent with prior studies in Zimbabwe and other sub‑Saharan African countries, which report that parental awareness, access to information, and health‑seeking behaviour significantly influence childhood immunisation coverage [6, 20]. Conversely, Marange apostolic affiliation was negatively associated with full immunisation, reflecting the persistent influence of religious and socio‑cultural beliefs on vaccine acceptance [10, 11, 21].

Health system barriers, including dissatisfaction with services, long waiting times, and stockouts, emerged as significant contributors to incomplete immunisation. Home births were associated with both partial and complete non‑immunisation, suggesting missed opportunities for early vaccination at birth [20, 22]. Collectively, these findings indicate that interventions to improve uptake must address both caregiver‑level factors (knowledge, perceptions, religious beliefs) and structural barriers within the health system. Integrated strategies involving community health workers, religious leaders, and targeted health education could strengthen vaccine coverage in rural and underserved populations.

Notably, partial and non‑immunisation may reflect different underlying dynamics: partial vaccination could indicate initial caregiver motivation followed by possible discouraging experiences with health services, whereas complete non‑immunisation may be driven by religion, cultural beliefs, or limited access [2, 20–24]. This distinction has important policy and practice implications. While broad health promotion may improve partial uptake, achieving full immunisation requires targeted interventions addressing home births, religious resistance, and maternal education [3, 4, 11]. These findings provide actionable evidence for the Ministry of Health, NGOs, and local health authorities to prioritise high‑risk groups, tailor community engagement, and ensure consistent vaccine availability.

### Limitations

The study has some notable limitations. Its cross‑sectional design precludes causal inference, and reliance on caregiver recall for information not captured in child health cards may have introduced recall bias. Findings may not be generalisable to urban or more socio‑economically advantaged populations. Some variables, such as distance to health facilities, were self‑reported and may be prone to measurement error.

### Recommendations

To enhance childhood immunisation coverage, health authorities should implement multifaceted interventions: strengthen health facility service quality, ensure consistent vaccine supply, engage religious communities to address socio‑cultural barriers, and promote caregiver health literacy through targeted education programmes. Special focus should be given to households with home births and children without health cards to reduce missed opportunities for vaccination.

## Conclusion

Childhood immunisation uptake in Gokwe South District remains suboptimal, shaped by maternal knowledge gaps, religious and cultural beliefs, and health system constraints. Interventions that simultaneously address caregiver education, service satisfaction, religious engagement, and systemic vaccine delivery barriers are likely to yield the most significant improvements in coverage. Distinguishing between predictors of partial and complete non‑immunisation can guide more tailored, effective strategies to achieve national and global immunisation targets.

## Data Availability

The data used and analysed during the current study contain individual‑level information that could potentially be identifiable. As such, the data are not publicly available due to confidentiality and privacy restrictions imposed by the authorising Ministry of Health. De‑identified data may be made available from the corresponding author upon reasonable request and subject to the necessary ethical and institutional approvals.
